# NHE Isoform Switching and KChIP2 Upregulation in Aging Porcine Atria

**DOI:** 10.1371/journal.pone.0082951

**Published:** 2013-12-23

**Authors:** Ritu Kant, Zhaoyang Hu, Jaideep K. Malhotra, Trine Krogh-Madsen, David J. Christini, Paul M. Heerdt, Geoffrey W. Abbott

**Affiliations:** 1 Bioelectricity Laboratory, Department of Pharmacology and Department of Physiology and Biophysics, School of Medicine, University of California Irvine, Irvine, California, United States of America; 2 Department of Anesthesiology, Weill Cornell Medical College, New York, New York, United States of America; 3 Department of Medicine, Weill Cornell Medical College, New York, New York, United States of America; 4 Department of Pharmacology, Weill Cornell Medical College, New York, New York, United States of America; Temple University, United States of America

## Abstract

Aging increases the risk of cardiac pathologies including atrial fibrillation and can alter myocardial responsiveness to therapeutic agents. Here, seeking molecular correlates of myocardial aging processes, we performed global “whole transcript” analysis of 25,388 genes using 572,667 probes to compare the left atrial (LA) transcriptomes of young adult (9 months old) versus elderly (10 years old) female swine. NHE2 (>9-fold) and KChIP2 (3.8-fold) exhibited the highest aging-related expression increases. Real-time qPCR recapitulated these findings and indicated a 50% decrease in LA NHE1, a twofold increase in right atrial KChIP2, but no significant changes for these transcripts in either ventricle. Notably, even in young adult pigs, NHE2 transcript was detectable and enriched in the atria over the ventricles. NHE1, the recognized cardiac isoform of the sodium hydrogen exchanger, has proven a compelling but clinically disappointing therapeutic target with respect to reperfusion arrhythmias. Our data challenge the dogma that NHE1 is alone in the myocardium and suggest that NHE2 could negatively impact the pharmacological responsiveness of atrial tissue to NHE1-specific inhibitors. KChIP2 is a cytosolic β subunit essential for generation of *I*_to_. The increased KChIP2 expression we observed with aging substantially shortened *in silico* atrial myocyte action potential duration, a predisposing factor in atrial fibrillation. Consistent with this, 4/5 elderly swine sustained pacing-induced AF≥15 s after cessation of stimulation, compared to 0/3 young swine. Our findings uncover potential molecular bases for increased arrhythmogenicity and reduced pharmacologic efficacy in the aging atrium, in a large animal model of human cardiac physiology.

## Introduction

Heart disease continues to be the leading global cause of mortality, accounting for one quarter of all deaths in the United States. Coronary heart disease (CHD), the most common form of heart disease, killed more than 400,000 United States citizens in 2008, while as many as 1% of the US population may suffer from atrial fibrillation (AF). While not solely a disease of the elderly, the risk of all types of heart disease, including AF, CHD and heart failure, increases with age [1]. The need for safe, effective therapeutics to combat cardiovascular diseases is therefore more important than ever, given the global increases in human longevity.

During and/or following ischemic episodes in the heart such as occur in CHD and heart failure, intracellular protons activate the cardiac myocyte sarcolemmal sodium hydrogen exchanger (NHE) to facilitate proton efflux and Na^+^ influx – causing, in turn, damaging cytosolic Ca^2+^ overload via the Na^+^/Ca^2+^ exchanger. Of the eleven known NHE isoforms represented in the human genome, NHE1 (also termed SLC9A1) is assumed to be the only cardiac-expressed isoform - NHE1 is expressed in the heart as well as other tissues, whereas the other isoforms are not considered to exhibit significant cardiac expression [2]. Numerous pre-clinical studies suggest that specific inhibition of NHE1 provides cardioprotection in ischemia/reperfusion injury, and cardiac hypertrophy/heart failure [3], [4]. Yet, clinical trials of NHE1-specific inhibitors (cariporide, eniporide and zoniporide) have been largely unsuccessful [5]. In patients with myocardial infarction (MI), despite encouraging data from a small-scale (100 patients) randomized trial of cariporide in patients with anterior MI who were administered reperfusion therapy via primary percutaneous transluminal coronary angioplasty (PTCA) [6], the larger-scale ESCAMI trial (1389 patients) indicated that eniporide does not reduce infarct size by attenuating reperfusion injury [7]. In patients at risk of MI, the large-scale GUARDIAN cariporide trial (11590 patients) was negative overall [8], although the highest cariporide dose was beneficial in high-risk coronary artery bypass graft (CABG) patients [9]. The subsequent EXPEDITION trial (2870 patients) showed that while cariporide reduced MI incidence, it significantly increased the rate of mortality associated with increased incidence of focal cerebrovascular events [10].

This major mismatch between the pre-clinical successes versus the clinical failure of NHE1-specific inhibitors has been a huge blow in the fight against cardiovascular disease. The debate over the mechanistic basis for this failure has centered around the timing of NHE1 inhibitor therapy, with two main hypotheses: that NHE1 inhibition needs to occur during ischemia (rather than just during reperfusion); and/or that inhibition during reperfusion is beneficial but that the timing of NHE1 inhibitor therapy did not allow for the drug to access the affected tissue immediately at the onset of reperfusion [5]. While timing appears a major factor, the debate continues and there is currently no resolution.

Interestingly, the aging human atrium was especially resistant to NHE1-targeted therapy in a recent study, performed using direct application of drug to atrial tissue *in vitro*, therefore alleviating concerns about tissue access by the drug. While cariporide was beneficial in a simulated ischemia model in protecting human atrial isolated from individuals <70 years old, it had no effect in the same study on atrial tissue isolated from patients >70 years old [11]. Aging also markedly increases the incidence of atrial fibrillation, a chronic rhythm disturbance that increases mortality from a number of other factors including stroke due to increased risk of clotting. An enhanced understanding of the aging-dependent remodeling of crucial genes orchestrating cardiac electrical excitability and contractility, including ion channels and transporters, will facilitate development of improved drugs tailored specifically to treat aging-related cardiovascular disorders.

While transgenic mice can provide fundamental insights into cardiac function and pathophysiology, they cannot faithfully model all aspects of human cardiac function, especially long-term changes during aging. However, molecular analyses of human subjects are hampered by high costs and difficulties in obtaining tissue from “healthy” individuals with relatively little inter-individual variability. Here, exploiting the very recent availability of the full *Sus scrofa* genome [12] to provide comprehensive transcript coverage, we utilized “whole transcript” transcriptome analysis to probe the expression of 25,388 genes in left atrial (LA) tissue from young adult versus elderly swine, an established large animal model of human cardiovascular physiology. We report aging-dependent expression differences in several genes, most strikingly those encoding sodium hydrogen exchangers (NHEs), with confirmatory real-time qPCR also indicating chamber-specificity of the remodeling. The results provide potential molecular bases for differential arrhythmogenesis and pharmacological responsiveness in elderly versus young adult myocardium, and suggest the hypothesis that NHE isoform switching in aging atrium might contribute to the clinical failure of NHE1-specific inhibitors.

## Materials and Methods

### Animal studies

After approval of the protocol by the Institutional Animal Care and Use Committee at Weill Cornell Medical College and in compliance with the National Institutes of Health “Guide for the Care and Use of Laboratory Animals”, eight female Sinclair swine, three of mean age 9±0.4 months (sexually mature) and five of mean age 122±4 months (last quartile of their expected lifespan of 12–13 years) (elderly) were used for the study. Given the homogeneity of age with each group, gender, and strain, animals were not formally randomized.

### Hemodynamic and electrophysiological data and tissue extraction

Following induction of anesthesia with intramuscular tiletamine/zolazepam (4.4 mg/kg) and xylazine (2.2 mg/kg), the trachea was intubated, and the lungs ventilated with oxygen and isoflurane to maintain end-tidal carbon dioxide at 32–35 mmHg and isoflurane at 1.3%. Following cannulation of a femoral artery for systemic blood pressure measurement and insertion of a pulmonary artery (PA) catheter via the right external jugular vein (for measurement of PA pressure), a median sternotomy was performed and the heart suspended in a pericardial cradle. A micromanometer catheter was inserted into the left ventricle via the apex for pressure measurement, and an electromagnetic flow probe placed around the ascending aorta for continuous measurement of cardiac output. Two small electrodes were applied to the LA appendage for recording of the epicardial atrial electrogram, for measurement of P-wave duration and half-time (the time required for peak amplitude to decline by half). Fine pacing wires were sutured to both atria, and the minimum output amplitude required for pacing capture (Medtronic 5328 Programmable Stimulator) was then determined. After recording baseline hemodynamic data, autonomic reflexes were blocked with a combination of hexamethonium (20 mg/kg, i.v) and atropine (2 mg/kg i.v.). Hemodynamic measurements were then repeated in the absence of sympathetic and parasympathetic nervous tone (Table 1). Differences between young and elderly animals were assessed with unpaired, 2-tailed student's t-test. Changes in hemodynamic variables produced by autonomic reflex blockade in each age group were assessed with paired, 2-tailed student's t-test. For all analyses, p<0.05 was considered significant. Finally, a stimulus was delivered at a cycle length of 75 ms for 10 seconds to induce AF, and the duration of maintained AF after cessation of pacing determined. Statistical significance for differences in AF induction between groups was compared using Chi^2^ analysis, with significance level set as p<0.05. With the animals deeply anesthetized, the heart was then arrested with iced potassium chloride solution and tissue samples flash frozen in liquid nitrogen for subsequent biochemical analysis. All efforts were made to minimize animal suffering.

**Table 1:** Hemodynamic parameters recorded from young and old swine used in the microarray and electrophysiological analysis.

	Baseline				Autonomic blockade			
	Young		Elderly		Young		Elderly	
	mean	SE	mean	SE	mean	SE	mean	SE
**HR (beats/min)**	106	4	84*	2	114¥	7	88*	2
**mAP (mmHg)**	83	4	76	2	81	2	67	5
**SVI (mL/m^2^)**	26.3	2.8	22.9	1.3	24.1	3.3	19.4	1.5
**CI (L/min/m^2^)**	2.80	0.39	1.92*	0.09	2.77	0.51	1.72*	0.17
**SVRI (dyn-sec-cm^−5^/m^2^)**	2266	426	3067	245	2292	475	3097	284
**mPAP (mmHg)**	17.1	2.2	16.9	1.3	17.7	2.2	15.5	1.2
**PVRI (dyn-sec-cm^−5^/m^2^)**	229	39	322	30	293	50	391	90
**LVPmax (mmHg)**	99	5	106	1	97	3	90¥	6
**LVEDP (mmHg)**	9.4	1.8	9.2	0.7	7.9	1.1	7.7	0.8

*n* = 3–5 pigs per group.

* Designates difference between age groups (unpaired t-test);

¥ designates a change produced by autonomic blockade within a group (paired t-test).

• HR = heart rate.

• mAP = mean arterial pressure.

• SVI = stroke volume indexed to body size.

• CI = cardiac output indexed to body size.

• SVRI = systemic vascular resistance indexed to body size.

• mPAP = mean pulmonary arterial pressure.

• PVRI = pulmonary vascular resistance indexed to body size.

• LVPmax = maximal left ventricular pressure.

• LVEDP = left ventricular end-diastolic pressure.

### Total RNA extraction

RNA was extracted using Trizol reagent (Invitrogen, Carlsbad, CA, USA). In brief, Trizol was added (100 mg tissue/1 ml) to the tissue samples (young, *n* = 3; old, *n* = 5) and homogenized on ice. Then, 0.2 ml chloroform/1 ml Trizol was added followed by centrifugation (4°C, 12,000 g, 15 min). Subsequently, the aqueous layer was carefully collected, 70% ethanol added, and RNA purified using the RNeasy Mini Kit (Qiagen, Hilden, Germany) according to the manufacturer's instructions. Total RNA was dissolved in 20–30 µL RNase-free water and stored at −80°C. The final RNA concentration and purity were assessed using the Nanodrop 2000 spectrophotometer (Thermo Scientific Inc, USA), with all samples having a 260/280 ratio of 2.0–2.3.

### Whole-transcript microarray analysis

Amplified and biotinylated sense-strand DNA targets from the entire expressed transcriptome were generated from the atrial RNA using the *WT Expression Kit* (Ambion) in conjunction with the *GeneChip WT Terminal Labeling Kit* (Affymetrix) according to manufacturers' instructions, hybridized for 20 hours on Porcine Gene 1.1 ST Array Strips (Affymetrix) then washed, stained and washed again with a *GeneAtlas* fluidics station (Affymetrix) before visualization and quantification of transcript expression on a *GeneAtlas* imaging station (Affymetrix). Each array contained 572,667 probes, interrogating 25,388 genes, giving a median 25 probes per transcript.

Statistical analysis of relative transcript expression and transcript annotation were performed using Partek Express software (Partek), with an RMA Import Normalization Algorithm [13] and Affymetrix library file, MO_PorGene-1_1-st-v1_strip_libraryfile. None of the samples failed quality control (QC), using default settings for Partek Express, which assess raw probe intensity mean, chip brightness, the mean of the median of the absolute residuals obtained from the model fitted in the probe set summarization step (to assess how well the probes fit the model for a chip), absolute relative log expression (RLE) across all of the probesets for each array (absolute RLE is obtained by calculating the absolute value of the log base 2 difference between the probeset signal estimate and the median of probeset signal estimates across all of the arrays; rle_mean is then calculated by taking the mean of absolute RLEs for all of the probesets on each array, with a low rle_mean indicating low biological variability in replicates). Principle component analysis (PCA) plot calculation [14] was performed with the correlation method. This is a 1-way ANOVA model by using Method of Moments [15]: Y_ij_ = μ+Strain_i_+ε_ij_, where Y_ij_ represents the jth observation on the ith Strain, μ is the common effect for the whole experiment, ε_ij_ represents the random error present in the jth observation on the ith Strain. The errors ε_ij_ are assumed to be normally and independently distributed with mean 0 and standard deviation δ for all measurements. The following contrast [16] was performed to compare genotypes; Log Transform Status was base 2.0, and *P* value, log fold change and ratios were calculated using geometric mean. Pathway analysis was performed using Pathway Analysis software (Ariadne Genomics).

### Real-time qPCR

cDNA was synthesized from 1 µg of RNA with Quantitect Reverse Transcriptase (Qiagen, Hilden, Germany) according to the manufacturer's protocol. To remove genomic DNA, template RNA was mixed with gDNA Wipeout Buffer and incubated at 42°C for 2 minutes. Quantitect Reverse Transciptase containing an RNase inhibitor and Quantiscript RT Buffer containing Mg^2+^ and dNTPs were then added to the genomic DNA elimination reaction and incubated at 42°C for 15 minutes. The reverse transcription reaction was inactivated with a 3-minute incubation at 95°C. Synthesized cDNA was analyzed immediately thereafter by qPCR or stored at −20°C until use.

Primer pairs for target gene *NHE1* (NCBI GeneID 397458) produced an amplicon of 241 bp. Primer pairs for target gene *NHE2* (NCBI GeneID 100101921) produced an amplicon of 252 bp. Primer pairs for target gene *KChIP2 (KCNIP2)* (NCBI GeneID 100037948) produced an amplicon of 250 bp. Primer pairs for reference gene *GAPDH* (NCBI Gene ID 396823) produced an amplicon of 697 bp.

Primer sequences for qPCR analysis were acquired from Primer3, version 4.0 and NCBI/Primer-BLAST. Primer sequences were as follows: *NHE1*, forward 5′- GGCTGCTACCTGTTCTCA -3′, and reverse 5′- AGGGAGATTTCGAAGGGTGT -3′; *NHE2*, forward 5′- GCGCGACATCACTTTGCTGGA -3′, and reverse 5′- GGGCCCACGACAAAGAAGTTG -3′; *KChIP2*, forward 5′- GACAGCGTGGAGGATGAGTT -3′ and reverse 5′- CATCGTGGTTGGTGTCAAAG -3′; and *GAPDH* forward 5′- GGCCCCCGTTCGACAGACAGC -3′ and reverse 5′- CGCAGCGCCGGTAGAAGCAGG -3′.

qPCR analysis was performed using the BioRad CFX Connect System with iTaq Universal SYBR Green Supermix (BioRad) and 96-well clear plates (BioRad). Each reaction contained ∼75 ng of cDNA, 1.5 µl of PCR-grade water, 1 µl of 10 µM forward primer, 1 µl of 10 µM reverse primer, and 5 µl of iTaq SYBR green, which was comprised of dNTPs, iTaq DNA polymerase, MgCl_2_, SYBR Green I, enhancers, stabilizers, fluorescein, and ROX normalization dyes.

Thermocycling parameters for BioRad's CFX Connect were as follows: for polymerase activation and DNA denaturation, 1 cycle at 95° (30 sec); for amplification, 35 cycles at 95° (5 sec) and 60° (30 sec); for melt-curve analysis, 65°C to 95°C with 0.5°C increment (2 sec/step).

Relative quantification was used to obtain normalized changes in expression levels of target genes (*NHE1, NHE2 and KChIP2*) relative to controls (*GAPDH*) using Bio-Rad CFX Manager Software Version 3.0. Each sample was run in triplicate as a quality control measure and triplicates varying from one another with a standard deviation of 0.300 or over were discarded and/or repeated. Melting curves were assessed for each reaction to verify the amplification of a single product. Final analysis of statistical significance was calculated measuring ΔΔCq values, with p<0.05 (unpaired, 2-tailed student's t-test) considered significant.

### Western blotting

Tissue samples were homogenized in PBS containing 30% (w/v) sodium dodecyl sulfate (SDS), using a pestle grinder system (Fisher Scientific, Hampton, NH, USA). Protein concentration was determined and normalized using the Bicinchoninic Acid (BCA) assay (Pierce Chemical Co. USA). Supernatants (50 µg protein/lane) were heated to 45°C and size-fractionated on 4–12% Bis-Tris gels (Invitrogen, NY, USA), transferred electrophoretically onto nitrocellulose membranes (VWR) and probed with mouse anti-KChIP2 K60/73 (UC Davis/NIH NeuroMab Facility) and mouse anti-GAPDH (Sigma) antibodies. Blots were washed and then incubated with goat anti-mouse immunoglobulin G (IgG) secondary antibody conjugated to horseradish peroxidase (Bio-Rad, CA, USA). Visualization of immunoreactive bands was performed by using ECL Western blotting reagents (Millipore, MA, USA) and signals visualized with the GBox imager (Syngene) and quantified with NIH ImageJ software 1.45S (Wayne Rasband, USA).

### Computer modeling

Simulations were performed using the recent human atrial myocyte model [17]. This mathematical model contains detailed formulations of sarcolemmal ionic currents and intracellular calcium and sodium ion fluxes. Because KChIP2 almost linearly controls I_to_ density [18], we modeled the 3.8-fold increase in KChIP2 as a 3.8-fold increase in I_to_ conductance. While other ionic currents (in particular the L-type calcium current) may be affected by KChIP2 upregulation, such effects are expected to be comparatively much smaller [18] and were not included here. The model was numerically integrated using a forward Euler method with an adaptive time step (between 1 and 10 µs).

## Results

### Hemodynamic measurements

At baseline, the only hemodynamic differences between groups were a lower heart rate and cardiac index in the elderly animals. These differences were maintained following autonomic blockade. Within age groups, autonomic blockade increased heart rate in young animals and decreased maximal left ventricular pressure in elderly pigs, but otherwise had no significant effect. However, these data need to be interpreted within the limitations of small sample size, and in consideration of the fact that all animals were deeply anesthetized.

### Microarray comparison of old versus young LA transcriptomes

**Figure 1:**
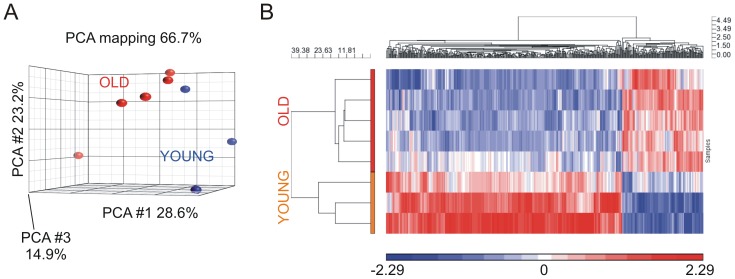
Whole transcript microarray of aging-associated remodeling in porcine left atrium. A. Principal component analysis (PCA) of global transcript expression changes in old (*n* = 5) versus young (*n* = 3) swine LA tissue. B. Heat map showing pattern of global transcript expression changes in LA isolated from old (*n* = 5) versus young (*n* = 3) swine (only genes showing ≥2-fold change between groups, P<0.05). Positive values (red) indicate genes increased in LA of old swine, negative (blue) indicates decreased expression in LA of old swine.

**Figure 2:**
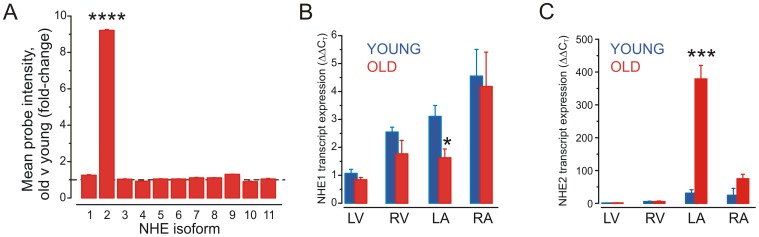
NHE isoform switching in aging porcine heart. A. Mean *NHE* isoform 1–11 transcript expression changes in LA of young versus old pigs as measured by microarray. ****p<2.0×10^−5^ old v young; other isoforms p>0.05; *n* = 3–5. B. Mean LA *NHE1* transcript expression quantified by real-time qPCR, standardized to GAPDH expression in each sample and then normalized to expression measured from a single young pig left ventricular sample for each gene. *p<0.05, old v young; all others p>0.05; *n* = 3–5. C. Mean LA *NHE2* transcript expression quantified by real-time qPCR, standardized to GAPDH expression in each sample and then normalized to expression measured from a single young pig left ventricular sample for each gene. ***p<0.001, old v young; all others p>0.05; *n* = 3–5.

Whole-transcript microarray analysis showed significant differences in the transcriptomes of young versus old pig LA (Figure 1 A, B) and revealed 94 transcripts with ≥2.0-fold increased expression in old versus young pigs. These were primarily metabolic enzymes, G protein-coupled receptor (GPCR) and non-GPCR cell-signaling genes, and genes related to inflammation (Table 2 and Spreadsheet S1 ). *NHE2* (also termed *SLC9A2*) showed by far the most statistically significant (p = 1.96×10^−5^) and greatest magnitude (9.2-fold) expression increase in LA of old versus young swine, with *KChIP2* (also termed *KCNIP2*) the next most up-regulated transcript (3.8-fold) (Table 3). Of the 78 transcripts down-regulated >2-fold in old versus young LA, the large majority (55) were GPCRs, mostly of no known function (Table 2, Table 3 and Spreadsheet S1). No significant changes in expression of the other ten *NHE* isoforms *NHE1* and *NHE3-11* were detected by microarray (Figure 2 A). Thus, the *NHE2* up-regulation was isoform-specific.

**Table 2:** Functional categories of transcripts with differential expression in left atrial tissue of old versus young swine.

Gene category	Increased in old LA	Decreased in old LA
GPCRs	9	55
Metabolic enzymes	28	8
Non-GPCR, cell signaling	16	7
Immune/inflammation	7	0
Solute carrier family (SLC)	3	1
Ion channel	3	0
Kinase	3	0
Cell motility	2	1
Extracellular matrix	2	1
Transcription factor	2	1
Microtubule dynamics	1	2
Other known functions	15	2
Unknown function	3	0
Total	94	78

Number of transcripts exhibiting expression changes with age, placed into functional categories. All transcripts with mean expression change ≥2-fold, P<0.05 between groups are included, after being filtered to remove transcripts with mean log 2 intensity <5 in both groups. Functional categorization based on literature search and/or or sequence homology. Functional categories with <3 total altered genes are grouped together in “Other known functions”.

**Table 3:** Transcripts showing statistically most significant LA expression differences between young and old swine.

Gene name	Protein product	Fold-change (Old v young)	p-value (Old v young)	F (Old v young)
LA expression increases with increased age				
*NHE2, SLC9A2*	Sodium hydrogen exchanger 2, NHE2	9.2	1.96×10^−5^	146
*TMEM90B*	SynDIG1	2.3	0.00014	73
*KChIP2, KCNIP2*	K^+^ channel interacting protein 2, KChIP2	3.8	0.000205	63
*CDHR1*	cadherin-related family member 1	3.5	0.000414	49
*ELMOD3*	ELMO/CED-12 domain containing 3	2.2	0.000579	44
LA expression decreases with increased age				
LOC100156333	OR 6C2-like	−5.9	0.000113	79
LOC100513280	OR 6C68-like	−5.3	0.000209	63
LOC100155907	OR 4Q2-like	−3.6	0.000787	39
LOC100513847	OR 7A17-like	−3.7	0.000806	39
LOC100516131	OR 4C16-like	−5.5	0.001048	35

The top 5 up- and down-regulated LA transcripts (comparing old to young swine) ranked by signal-to-noise ratio (F). OR = olfactory receptor and indicates a putative orphan GPCR (by sequence homology). Before ranking, transcripts with mean expression change <2-fold, P≥0.05, or with mean log 2 intensity <5 in both groups, were filtered out.

### Aging-dependent changes in atrial and ventricular NHE2 and KChIP2 expression

**Figure 3:**
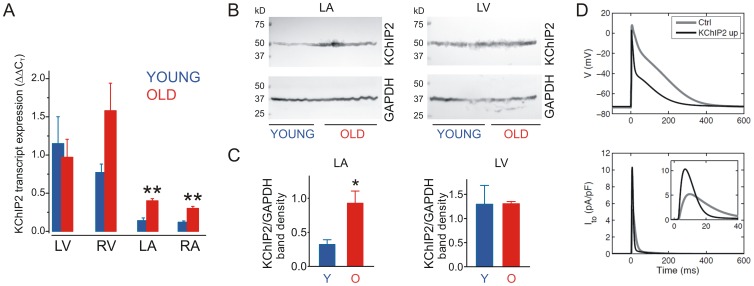
KChIP2 upregulation in aging porcine atria. A. Mean KChIP2 (*KCNIP2*) transcript expression quantified by real-time qPCR, standardized to GAPDH expression in each sample and then normalized to expression measured from a single young pig LA sample. **p<0.01, old v young; all others p>0.05; *n* = 3–5. B. *Upper*: mean cardiac KChIP2 protein expression visualized by western blotting, standardized by total protein for loading. *Lower*: GAPDH expression for comparison. Each lane is from a different individual. C. Mean cardiac KChIP2 protein expression quantified by band densitometry of blots as in panel F, normalized to GAPDH expression. *p<0.05 old v young; *n* = 3–4. D. *In silico* modeling data showing the predicted effects of 3.8-fold KChIP2 upregulation on human atrial myocyte *I*_to_ (*lower*) and action potential morphology (*upper*).

**Figure 4:**
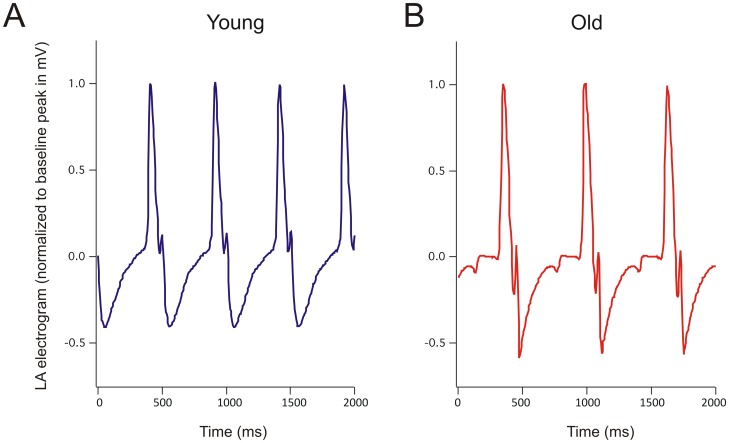
Atrial electrophysiology of young versus aging swine. A. Exemplar atrial electrogram recorded from a young female pig. B. Exemplar atrial electrogram recorded from an old female pig.

Real-time qPCR verified the robust LA up-regulation of NHE2 transcript expression in aging pigs (p = 0.0009), indicated a trend toward increase (p = 0.09) in old versus young right atrium (RA), and no difference in ventricular *NHE2* expression with aging. Of note, *NHE2* was enriched in the atria compared to ventricles in both age groups, and readily detectable even in young pig atria. NHE1 was also enriched in atria over ventricles and relatively invariant with aging although real-time qPCR detected a 50% decrease in expression in old versus young LA. This divergence from the whole transcript microarray data is suggestive of alternate splicing of this gene in aging (Figure 2 B). Real-time qPCR indicated a >twofold increase in *KChIP2* transcript expression in aging in the LA, RA and right ventricle (RV), although in the latter it did not reach statistical significance (Figure 3 A). KChIP2 protein expression was increased threefold in aging compared to young adult pig left atrium, but unchanged in left ventricle (Figure 3 B, C). *In silico*, in a human atrial myocyte model [17], the increased KChIP2 expression increased *I*_to_, shortening action potential duration primarily from faster phase 1 and 2 repolarization (Figure 3 D). Representative epicardial LA electrograms are shown in Figure 4. We did not detect statistically significant age-related differences in LA P-wave duration (young 107±9 ms, elderly 123±7 ms, p = 0.22) or half-time (time to 50% decline from peak amplitude) (young 29±4 ms, elderly 33±5 ms, p = 0.65). However, in 4/5 elderly animals, AF continued for at least 15 s after cessation of burst pacing, compared to 0/3 young animals (p<0.05).

## Discussion

Our results, obtained from an unbiased whole-transcript microarray analysis of the majority of known genes in the newly-sequenced porcine genome, uncover striking aging-related changes in various genes; here we focus on the two for which we found the highest left atrial transcript expression increase in aging – *NHE2* and *KChIP2*. Firstly, the data suggest, surprisingly, that *NHE1* is not the only *NHE* isoform expressed in the atria even in young adults, at least in this large animal model. NHE inhibitors previously tested in clinical trials, namely cariporide, eniporide and zoniporide, are 30- to 200-fold less potent inhibitors of NHE2 than NHE1; therefore the doses used in clinical trials would not be predicted to substantially inhibit NHE2. If NHE2 up-regulation were contributing to pathology in ischemia/reperfusion injury in patients in the clinic, it would not have been targeted because the therapies were all aimed at the more drug-sensitive isoform, NHE1. NHE2 also behaves differently to NHE1 in various aspects, including response to Ca^2+^, K^+^, regulatory proteins and transcriptional activators [19]. Indeed, we do not know exactly how NHE2 functions when expressed in cardiac myocytes because there has not previously been a compelling reason to study this. NHE isoform-switching in aging individuals (the more vulnerable and at-risk population for ischemia-reperfusion injury and heart failure) could lead to fundamental changes in therapeutic targeting of NHE in cardiac injury and heart failure. However, despite the potential significance of this finding and possible relevance to cardiac function and pharmacology, especially in ischemia reperfusion injury, it is important to note that we do not yet have direct evidence regarding the relationship between cardiac NHE2 expression and its increase in aging, and altered function or pharmacology of aging tissue.

KChIP2 upregulation is predicted to shorten the action potential because it acts as a positive regulator of Kv4 α subunits, promoting their surface expression and thus augmenting the early repolarization current, *I*_to_. Increased atrial *I*_to_ with aging could predispose to AF (a predisposition observed here in aging swine), while increased ventricular *I*_to_ is linked to Brugada syndrome because it diminishes the depolarizing force of the voltage-gated sodium current. In mice, *KChIP2* deletion removes *I*_to_ completely and predisposes to ventricular tachycardias during rapid pacing [20], but as mice rely much more on *I*_to_ for repolarization because of their high heart rate, this finding is not necessarily entirely translatable to human heart.

**Figure 5:**
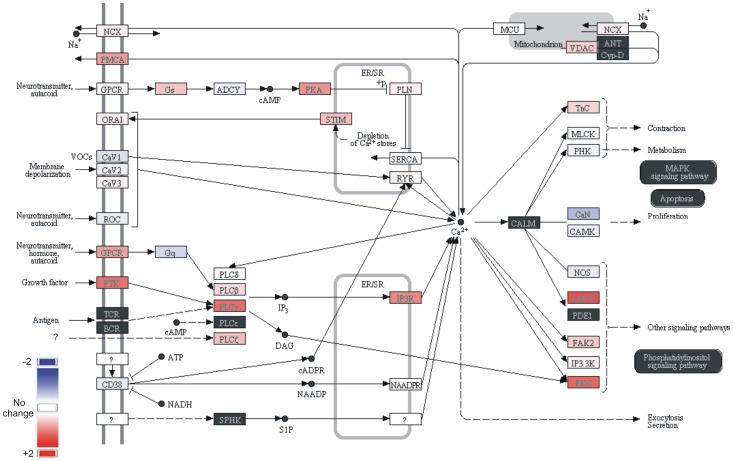
Calcium signaling network remodeling in aging porcine left atrium. Differentially expressed genes within a calcium signaling network in old versus young left atrium; network identified by pathway analysis (Ariadne). p = 0.009 for old versus young left atrial tissue with respect to the gene network shown (*n* = 3–5).

This brings us to the limitations of the present study – our findings are striking but will need to be repeated with human heart in a manner that controls as much as possible for variations other than age, and analyses equivalent regions between groups, taking into account factors such as the strong transmural gradient of *KChIP2* observed in previous studies. Also, a follow-up study in swine with larger group sizes might facilitate more definitive functional analyses than was possible here. A further limitation is that with existing commercial NHE antibodies we were unable to generate protein expression data for NHE isoforms in pig heart. As important as future human expression analyses, will be functional studies in experimental models to assess the role, if any, of atrial NHE2 expression in determining the pharmacology of cardiac sodium/hydrogen exchange, particularly in the context of reperfusion arrhythmias. Furthermore, additional studies are required to understand the potential significance of our observed aging-increased expression of transcripts for cadherin, and whether this alters, e.g., the formation of adherens junctions in aging atrium; also that of changes in SynDig1 and ElmoD3 and other upregulated transcripts. Finally, the significance of the striking aging-dependent atrial downregulation of many orphan GPCRs will not be appreciated until their roles in the heart are discovered. These GPCRs might be involved in aspects of myocyte calcium signaling networks, statistically significant changes in aging-associated changes in which were detected using pathway analysis software (Figure 5).

## Supporting Information

Spreadsheet S1 Differentially expressed gene transcripts in old versus young pig atria (>2-fold, P<0.05) revealed by microarray analysis.
(XLSX)
